# The positive effects of combined breathing techniques and cold exposure on perceived stress: a randomised trial

**DOI:** 10.1007/s12144-022-03739-y

**Published:** 2022-10-07

**Authors:** Cristopher Siegfried Kopplin, Louisa Rosenthal

**Affiliations:** 1 Chair of Marketing & Innovation, Universitätsstraße 30, 95447 Bayreuth, Germany; 2 University of Bayreuth, Bayreuth, Germany

**Keywords:** Perceived stress, Breathing technique, Cold exposure

## Abstract

A pranayama-inspired breathing technique, cold exposure, and their combined application were assessed for their potential to reduce perceived stress in adults and compared to a control group. An experiment involving four groups was conducted, yielding separate cells for breathing technique-only and cold exposure-only, as well as a combined treatment and a control group. Eighty-six individuals participated in the study. Perceived stress is measured employing the 10-item version of the Perceived Stress Scale (PSS-10) and the 20-item version of the Perceived Stress Questionnaire (PSQ). The instruments exhibit a substantial correlation (r = 0.842, p < 0.001). The combined group exhibited a medium to large positive effect on perceived stress compared to the control group. The breathing technique and cold exposure on their own were not found to yield substantial effects, indicating synergies between both exercises. Combinations of breathing techniques and cold exposure may be employed to decrease individuals’ perceived stress.

## Introduction

Stress is a major concept regarding individual health. Perceived stress is a widely employed notion to capture individuals’ subjective stress experience (Cohen, 1988; Fliege et al., 2005; Roberti et al., 2006; Taylor, 2015). This subjective evaluation is a relevant part of an individual’s response to stress (Cohen et al., 1983). Experience of stress is associated with a variety of negative outcomes such as a neglect of physical exercise, increases in drinking and smoking, and the adoption of an unhealthy diet in terms of high fat intake (Leventhal et al., 2017; Mikolajczyk et al., 2009; Ng & Jeffery, 2003; Vidal et al., 2018). Other adverse consequences include depression (Spada et al., 2008), anxiety (Shi et al., 2020), increased levels of inflammation, and a heightened risk of cardiovascular disease (Black & Garbutt, 2002; Richardson et al., 2012). Hence, it is important to provide remedies that attenuate this downward spiral.

Simultaneously, recent years have witnessed the uprising of the Wim Hof Method (WHM), a combination of pranayama-like breathing and cold exposure in the form of cold showers and, in its more extreme variant, ice baths. These two components are complemented with exercises seeking to increase practitioners’ commitment, such as push-ups, stretching, and a reduced number of yoga poses (Innerfire, 2021), which are used to build the discipline for regular application of breathing techniques and cold exposure. Previous research has focused on physiological measures such as inflammation markers and the impact on inflammation-related illnesses such as arthritis (Buijze et al., 2019; Zwaag et al., 2020), altitude acclimatization (Buijze & Hopman, 2014), and the activity of the autonomic nervous system (Kox et al., 2012, 2014). We seek to contribute to the body of knowledge by highlighting the WHM’s association with reduced perceived stress.

The remainder is structured as follows. Section 2 details related work on the matter. The experiment design is explained in Sect. 3, followed by the results in Sect. 4. Findings are discussed in Sect. 5. The paper closes with concluding remarks in Sect. 6.

## Related work

### Perceived stress

An individual’s response to a stressful event is “not based solely on the intensity or any other inherent quality of the event, but rather is dependent on personal and contextual factors as well” (Cohen et al., 1983). Researchers have emphasized not to focus on objective stress only but to capture its appraisal, which is critical for the inception of psychological stress (Muzik & Diwadkar, 2019; Schiffrin & Nelson, 2010). This inception rests upon the two components of cognitive appraisal and affective integration (Everly & Lating, 2019; Kleckner et al., 2017; Muzik & Diwadkar, 2019). Cognitive appraisal comprises the meaning that an individual assigns to an event and an individual’s assessment of their available resources to handle it (Barrett, 2012; Everly & Lating, 2019; Schneider et al., 2020). If these resources are evaluated to be insufficient, coping fails, and perceived stress emerges (Cohen et al., 1998; Lazarus & Folkman, 1984; Schneider et al., 2020). Affective integration denominates emotional arousal linked to the cognitive appraisal (Everly & Lating, 2019). Emotional arousal is primarily induced in the limbic system of the brain including the insula (Barrett, 2012; Muzik & Diwadkar, 2019). If the two components come together to form perceived stress, this reaction may adversely affect physiological and mental well-being (Lazarus & Folkman, 1984; Schneider et al., 2020).

Perceived stress and, worse, unmanaged perceived stress, is a common issue across modern countries (Mathur et al., 2016) and has been increasing over the last decades (Cohen & Janicki-Deverts, 2012). Adverse consequences may include a proclivity for unhealthy behavior, such as smoking, drinking alcohol, neglecting physical exercise, and adopting a high-fat and low-fruit and low-vegetable diet, and addictive behavior such as excessive smartphone usage, depending on an individual’s success in mood self-management (Leventhal et al., 2017; Liu et al., 2018; Mikolajczyk et al., 2009; Ng & Jeffery, 2003; Vidal et al., 2018). Further outcomes of perceived stress include anxiety (Shi et al., 2020), depression (Spada et al., 2008), and post-traumatic stress disorder (Wang et al., 2019) but also more physiological outcomes such as elevated pro-inflammatory mediators and an increased risk of cardiovascular disease (Black & Garbutt, 2002; Cutolo et al., 2000; Richardson et al., 2012; Wright et al., 2019).

Recently, major events such as the outbreak of the COVID-19 pandemic have highlighted the importance of understanding and managing perceived stress (Liu et al., 2021; Yan et al., 2021; Zhao et al., 2021). Findings particularly illuminate an association between perceived stress and depression (Cristóbal-Narváez et al., 2020; Hewitt et al., 1992; Liu et al., 2021), which is a relevant insight as depression is linked to suicidality (Bergfeld et al., 2018; Dold et al., 2018), and individuals tend to seek information online and in isolation instead of reaching out for medical attention (Solano et al., 2016). Hence, it appears that reducing perceived stress is an early intervention in the context of multiple adverse health outcomes.

Several contemplative practices have been suggested to improve well-being under these circumstances, such as controlled breathing as a part of yoga or the WHM (Agarwal et al., 2020). Scholars find that “[e]motions and respiration are closely linked in a complex feedback loop” (Jerath et al., 2015), indicating that techniques employing breath control may be a promising venue to lower perceived stress. Indeed, empirical findings show that yoga practices consisting of physical postures, meditation, and breathing techniques as well as breathing alone decrease perceived stress (Cowen, 2010; Kanchibhotla et al., 2021; Lei Chui et al., 2021; Naik et al., 2018).

### Benefits of cold exposure and breathing techniques

Signals of either physical stress (such as heat or cold) or psychological stress trigger the same response mechanisms (Selye, 1976). This response can increase the organism’s performance to cope with the perceived stress and protect itself, and further seeks to learn from the information to efficiently tackle similar situations in the future (Godoy et al., 2018; Ulrich-Lai & Herman, 2009), given that the exposition to stress is neither too long nor too intense (Le Bourg, 2020). Hence, it appears plausible that experiencing one class of stress (e.g., physical stress) may support coping with future instances of the other class (e.g., psychological stress).

Research has examined the potential of mindfulness-related techniques such as yoga, particularly the influence of yogic breathing, i.e., pranayama (Cowen, 2010; Kanchibhotla et al., 2021; Lei Chui et al., 2021). Extant findings show that these exercises can improve markers that are symptomatic for perceived stress, such as inflammatory mediators (Kox et al., 2014; Zwaag et al., 2022). An important concept in research on physiological stress countermeasures, explaining their mechanisms of action, is that of hormesis. Hormesis, i.e., moderate stressful impact, yields positive effects on the immune system and adaptation, while high stress levels have the opposite effect (Calabrese & Baldwin, 2003; LaVoy et al., 2011; Mattson, 2008; Stebbing, 1982), suggesting an inverse u-shaped relationship between stress and supporting effect. These moderate doses allow an adaptation and, therefore, improved responses to future stress (Calabrese & Agathokleous, 2022; Le Bourg, 2020; Schirrmacher et al., 2021).

One source of hormetic stress is an individual’s surroundings, such as heat and cold, respectively. When examining different types of environments, e.g., water or air, according to their hormetic potential, water temperatures below the threshold of 20°C (68°F) may be considered cold (LaVoy et al., 2011). This threshold corresponds with cold exposure found in health-related applications such as cold showers, which commonly range between 10 and 14°C (50 to 57.2°F) in temperature (Buijze et al., 2019).

Exposure to such environments is stressful – without further need for physical exercise – and evokes the release of stress hormones (LaVoy et al., 2011). When exposed to cold, the body reacts on a physiological level in the form of shivering thermogenesis and peripheral vasoconstriction, i.e., involuntary muscle contraction and restrictions of blood flow to the skin (Haman & Blondin, 2017; LaVoy et al., 2011). Consequently, blood volume is allocated to the core. Research shows that hormetic stress induced through showers and baths (e.g., balneotherapy and hydrotherapy, both of which may be briefly described as therapeutical interventions using water) yields attenuating effects on inflammation and further stress-related outcomes (Gálvez et al., 2018). In hydrotherapy, which is relevant for the study at hand, only the physical qualities of the water such as its temperature yield an impact, as opposed to biochemical components applied in balneotherapy (Gálvez et al., 2018). Thus, the cold exposure element of the WHM, which focuses on cold showers and – if the practitioner wishes to do so – ice baths (Innerfire, 2021), constitutes a form of hydrotherapy.

As a result of hormesis, the sympathetic nervous system is stimulated (LaVoy et al., 2011); a part of the nervous system which is typically considered an autonomous part that is inaccessible to voluntary manipulation. Recent findings, however, suggest that the sympathetic nervous system might also be activated using techniques such as the breathing technique from the WHM (Kox et al., 2014; Muzik et al., 2018; Zwaag et al., 2020) or yogic breathing, i.e., pranayama (Jerath et al., 2006; Shapiro et al., 2007; Telles et al., 1994). Here, it is interesting to note that the breathing technique of the WHM is similar to pranayama (Agarwal et al., 2020; Kiecolt-Glaser et al., 2010), and both appear to yield congruent effects: the activation of the sympathetic nervous system leads to increased levels of epinephrine and a diminished innate immune response, which is expected to attenuate inflammation as is the case with autoimmune diseases (Kox et al., 2014; Zwaag et al., 2020, 2022). Indeed, early empirical evidence suggests an alleviating influence on inflammation in the case of arthritis (Buijze et al., 2019).

### WHM as an integrated mindfulness application

Research has adopted the notion of mindfulness and its potential attenuating role for stress and stress-related outcomes (Liu et al., 2018). Indeed, findings suggest a positive impact of mindfulness on perceived stress (Baer et al., 2012; Bao et al., 2015). Although frequently associated with meditation, the term describes a state of consciousness where the individual is focused on their moment-to-moment experience (Brown & Ryan, 2003; Shapiro et al., 2006). Thus, meditation is a vehicle to achieve mindfulness, while the state may be reached through other means as well. Mindfulness has been found to suppress emotional and behavioral automatisms and improve adaptation to adverse stimuli (Lueke & Gibson, 2015; Shapiro et al., 2006). Hence, it is assumed to “buffer the undesirable impacts of a negative factor” (Liu et al., 2018).

**Fig. 1:**
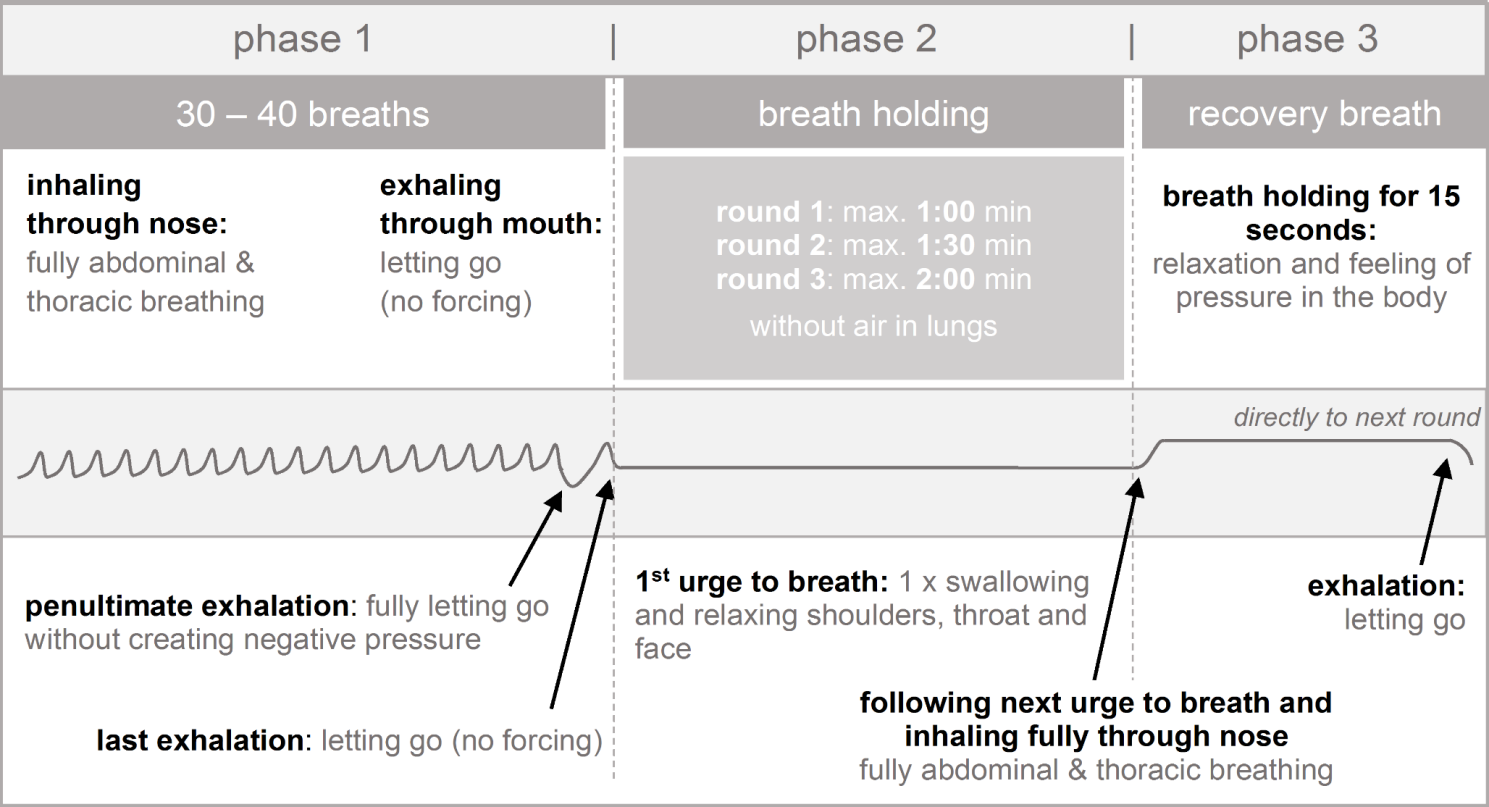
WHM breathing technique

Within the WHM, breathing techniques and cold exposure aim at both directly eliciting physiological reactions and calming the practitioner’s mind (Innerfire, 2021). Thus, in total, the WHM consists of three pillars that are designed to interact with each other: breathing, cold therapy (i.e., cold exposure), and commitment (Innerfire, 2021). The breathing technique combines hypoxic and hyperoxic phases. The exercise starts with 30 to 40 deep nasal inhalations involving abdominal and thoracic breathing and oral exhalation. The exhalation breath should be performed as “letting go” of the air without forcefully pressing it out. After the last exhalation, the individual holds their breath. For a regular WHM practitioner, breath holding time may be stretched out at will. For the context of this manuscript, we set specific round limits of 60, 90, and 120s for the three rounds. Once this time is passed, the individual takes a deep breath and holds it for 15s before letting go. This step completes one round of the breathing exercise. Figure1 shows the complete process. The hyperventilation in phase 1 evokes respiratory alkalosis at the beginning of phase 2 (Djarova et al., 1986; Muzik & Diwadkar, 2019).

Cold exposure is commonly implemented in the form of cold showers but may also encompass taking an ice bath or putting one’s hand or face into a bowl of ice. The goals of this exposure are the creation of brown fat, the reduction of inflammation, increased hormone balance, and elevated endorphin levels (Innerfire, 2021). The third element, commitment, is viewed as an enabler or foundation, as it seeks to help to build up patience and focus for the successful application of the other two pillars, and is associated with patience, dedication, and willpower (Innerfire, 2021). Actively working on one’s commitment may incorporate physical exercises such as headstands, front splits, and stretching.

Extant research has investigated the effects of the WHM. Studies have found anti-inflammatory impacts (Buijze et al., 2019; Zwaag et al., 2020), control over autonomic parts of the nervous system (Kox et al., 2012, 2014; Muzik et al., 2018), resistance to endotoxins (van Middendorp et al., 2016), and accelerated acclimatization to high altitudes (Buijze & Hopman, 2014). Consistent with the WHM’s notion of three integrated pillars, empirical evidence shows that the main techniques of breathing exercises and cold exposure may yield unique physiological effects (Zwaag et al., 2022). Hence, it may be expected that their combined application leads to an interaction and thus, greater effects than a total of the two isolated techniques would suggest. Consequently, the study at hand seeks to test the following hypotheses:

H1. Breathing techniques are associated with decreased levels of perceived stress.

H2. Cold exposure is associated with decreased levels of perceived stress.

H3. Levels of perceived stress are lower for combined approaches integrating breathing techniques and cold exposure than for single-method approaches.

## Experiment design

### Groups and treatments

A between-subjects design with one factor and four levels was used. One group was employed as a control group and did not receive any form of training. For the treatment, three groups were distinguished: one group only performed daily breathing exercises (group ‘breathing technique’), another conducted cold exposure in terms of having a cold shower every morning (group ‘cold exposure’), and the third treatment group undertook both (group ‘combined’). Morning sessions were defined as the creator of the WHM himself suggests exercising on an empty stomach before breakfast (Innerfire, 2021), and extant research identified significant differences between morning and afternoon sessions regarding blood parameters (Zwaag et al., 2022).

The experiment was conducted over 14 days. Perceived stress was measured before (t_0_) and after the intervention (t_1_) using the PSS-10 and the PSQ instruments to compare pre- and post-treatment levels and between-subject differences. Participants tracked their daily exercises to provide information about successful experimental manipulation. In line with the extant literature, participants were instructed by trainers of the WHM (Buijze et al., 2019; Kox et al., 2014). Recent research has shown that the exercises can be learned in a short time frame and are performed adequately (Zwaag et al., 2022). The individuals were assessed for health issues and demanded to seek medical advice before participation. Sample size was determined employing a significance level of 0.05, an estimated statistical power of 0.80, and an effect size of 0.25, resulting in a total sample size of 100.

#### Cold exposure

Consistent with previous studies, cold exposure was implemented in the form of cold showers, yielding about 10 to 14°C (50 to 57.2°F, Buijze et al., 2019). To count as a cold shower, the temperature needed to be lowered as far as possible with the respective control. Showers were taken at home every morning and the exposure time was increased throughout the experiment. For both groups that employed cold exposure, the instruction demanded a cold shower per day. Participants were asked to track their progress. The cold exposure treatment was composed of five steps: three minutes of the horse stance exercise to warm up, followed by the individual’s regular (warm) shower. To prepare for the cold exposure mentally, participants were asked to take ten deep breaths. To gradually increase the cold exposure, the length of the cold shower started at 15s and was increased to a maximum of 60s. The cold shower may be taken separately or follow a regular warm shower taken for personal hygiene. Head and neck were not covered by the cold water. Participants were instructed that after taking the shower, they should warm up by moderate physical exercise. The WHM recommends the “horse stance”, a pose known from martial arts, which is similar to a squat with the legs being opened a little more. Hence, the participant in the horse stance appears like a rider in the saddle. After the shower, the horse stance exercise was repeated for three minutes to warm up again.

#### Breathing technique

The implementation of the breathing technique was similar to the study by Buijze et al. (2019), although with shortened durations for each step. Figure 1 illustrates the technique. One round of the breathing exercise comprised of 30 deep breaths, followed by exhalation and breath-hold. Participants were instructed to continue breathing when (1) they felt a stimulus to do so or (2) when a safety time limit was passed. After this phase, a deep inhalation breath was taken, and the breath was held again (i.e., recovery breath), this time for 15s. This procedure was repeated a total of three times. The safety time limit was 60s for the first round, 90s for the second, and 120s for the third round. Participants were asked to perform the breathing technique on an empty stomach at the start of the day. Individuals in the full treatment (i.e., ‘combined’) group were recommended to take the cold shower after breathing, as the exercise helps to commit to the cold exposure.

### Assessment

Perceived stress was measured using two established instruments, the PSS-10 (Cohen, 1988) and the revised 20-item version of the PSQ (Fliege et al., 2005). Both are established and validated instruments (Fliege et al., 2005; Levenstein et al., 1993; Makhubela, 2020; Roberti et al., 2006; Smith & Emerson, 2014; Taylor, 2015). In line with previous research, the positively worded items of the PSS-10 (items 4, 5, 7, and 8) are reverse coded (Reis et al., 2017). Thus, high scores indicate higher levels of perceived stress, and low scores depict lower levels of perceived stress. For the PSQ, the positively formulated items are reverse coded as well, with the same interpretation. Appendices A and B list the items. Data may be acquired from the authors upon request.

### Participants and instructions

Participants were recruited via social media, posting ads in blogs related to health, fitness, and mindfulness. Participants suffering from health conditions that are listed as contraindications for the WHM were excluded. Table1 details inclusion and exclusion criteria. Further, participants were requested to seek medical counsel before the experiment to ensure exclusion criteria are respected. Participants were assigned to the groups via block randomization.

**Table 1:** Inclusion and exclusion criteria.

Inclusion criteria	Exclusion criteria
Legal age	Epilepsy Hypertension Coronary heart disease Heart failure Stroke Pregnancy Type 1 diabetes Asthma Diagnosed mental illness, e.g., depression* Intake of antidepressants Intake of beta blockers Intake of ACE inhibitors

*Note that diagnosed depression without medical treatment was an exclusion criterion

Ninety-nine participants were recruited, 61 of which were female, 29 were male, and 9 indicated a diverse gender. Age ranged from 21 to 56, with a median of 29 (M = 31.36, SD = 7.98). Most participants had a German nationality (n = 76). Regarding the personal environment, which may impact perceived levels of stress, the majority of participants lived with their partner (n = 48), followed by participants living alone (n = 16) or with roommates (n = 15). Eleven participants, ranging between 21 and 29 years of age, reported that they lived with their parents. Considering education, most participants graduated from university (n = 62), followed by matriculation standard (n = 19). Fourteen participants indicated a lower education level, and 4 abstained from a response. Thirty-eight worked full-time, and 15 participants reported to be freelancers, while 14 worked part-time, and 13 were university students.

To ensure that all subjects receive the same instructions, a one-day introductory course was held led by a certified WHM instructor. The session was implemented as an online conference. Participants were free to ask questions and were corrected and guided during the session when necessary. Afterward, they were introduced to the training schedule that they were expected to follow during the experiment. For the breathing technique group, the instruction was started with an introductory exercise that illustrated the participants’ normal breathing to create awareness for the correct technique of the WHM. Three guided rounds of the breathing technique were conducted. For the cold exposure and the combined group, the cold shower was detailed, and the horse stance exercise was explained and trained.

Participants were instructed to perform the exercise on an empty stomach before breakfast and were handed a protocol sheet to fill in each exercise for the 14-day period. For the breathing technique and the combined group, a copy of Fig. 1 was provided to serve as a reminder of the correct process. For the cold exposure and the combined group, the protocol also contained an explanation of the horse stance exercise. The documentation was checked after seven days (i.e., the first half of the trial) and after 14 days.

### Measures to ensure validity

#### Experimental mortality

To prevent participants from dropping out due to the unpleasant experience of cold water, they were advised to conduct the horse stance exercise afterward if they felt cold. Participants in the full treatment group were recommended to take the cold shower after the breathing technique, as it is supposed to increase the practitioner’s mindfulness and commitment and decrease the activity of pain receptors, which facilitates the cold exposure.

#### History

The experiment took place in June 2021 during the COVID-19 pandemic. At this point, the pandemic had been part of daily life for about a year and a half. Thus, it is expected that the study’s participants were used to the altered circumstances. To rule out distorting effects on the experimental manipulation, a number of control questions were implemented to assess whether COVID-related influences had an impact on the participants’ perceived stress level.

## Results

### Baseline analyses

#### Initial levels of perceived stress

Perceived stress was measured before (t_0_) and after the experiment (t_1_). In t_0_ (n = 99), the PSS-10, as well as the PSQ scores, did not differ between the groups based on an ANOVA (F(3, 98) = 0.283, p = 0.837). Hence, we may assume the absence of a systematic difference. For PSS-10, initial mean values were 2.85 (SD = 0.622) for the control, 2.91 (SD = 1.01) for the breathing technique, 2.89 (SD = 0.84) for the cold exposure, and 2.72 (SD = 0.73) for the combined group.

For the PSQ, similar results of no significant differences were found (F(3, 98) = 0.746, p = 0.527). The mean scores were 2.38 (SD = 0.44) for the control, 2.41 (SD = 0.76) for the breathing technique, 2.43 (SD = 0.52) for the cold exposure, and 2.21 (SD = 0.62) for the combined group.

#### Participant expectations

Several questions were used to measure potential expectations regarding the effects or outcome of the study. Participants were asked whether they had any expectations at all, if these were positive, neutral, or negative, and whether their expectations turned out to be correct. After the intervention, participants were also interviewed regarding their content with the procedures, as perceptions of an unpleasant experience may yield distorting effects on the perceived stress variables. Cross-tabulation was used to compare participant’s expectations regarding the study. Employing χ² tests, no heterogeneity was found, indicating that expectations were rather similar among the individuals. Thus, we assume that they do not threaten our analysis.

#### Distorting Environmental Effects

To ensure that environmental conditions such as housing, marital status, and income do not account for perceived stress, correlation analyses were conducted. The absence of significant correlations with PSS-10 and PSQ scores was used to assume that these conditions do not threaten the results. Sociodemographic data was compared across the groups via ANOVAs (metric data such as age) and χ² tests (nominal data such as gender), indicating the absence of significant differences.

#### History

We used measures for alternative explanations to ensure the effects may be attributed to the treatment. No group differences were found regarding reported impacts by COVID-19, potential changes in the personal environment, and concerns regarding the exercises based on χ² and Fisher tests. Hence, it is assumed that differences in perceived stress levels can be attributed to the experimental manipulation.

### Safety

Adverse events (AEs) and severe adverse events (SAEs) were assessed. In total, five AEs occurred. Three participants suffered from pollen allergy, and another participant reported a dry throat, which impeded their execution of the breathing technique. One participant in the combined group skipped one day of cold exposure due to having been vaccinated. No SAEs were recorded.

### Descriptive statistics of the final sample

A small number of participants dropped out throughout the experiment: four participants did not attend the instruction (one from the breathing group, one from cold exposure, and two from the combined group), and two additional participants abandoned treatment (P_1_ indicated a lack of time, and P_2_ could not overcome their aversion regarding the cold shower). In sum, 93 participants completed the experiment. Additionally, manipulation check evaluation demanded the removal of seven data points. These seven participants performed the instructed techniques on less than 10 out of 14 days. The removed data points stemmed from the breathing technique group (n = 3), cold exposure (n = 1), and the combined group (n = 3). More strict data cleansing, i.e., restricting the sample to participants exercising 11, 12, 13, and 14 out of 14 days, respectively, did not change the results in terms of significance. Thus, for more statistical power, the initial choice of 10 days was maintained as a threshold. The excluded data points stemmed from all intervention groups, with the combined group yielding the largest proportion (control = 0, breathing technique = 2, cold exposure = 1, combined = 4). The final sample consists of 86 observations, distributed rather equally across the four groups: breathing technique-only (n = 20), cold exposure-only (n = 22), combined group (n = 18), and control group (n = 26).

### Repeated measures ANOVA

As perceived stress was measured at two points in time – to assess baseline values and after the intervention – an analysis of variance for repeated measures (repeated measures ANOVA) approach was employed for hypothesis testing. The analysis run begins with an assessment of the PSS-10. Cronbach’s Alpha yielded a satisfying value of 0.802 for internal consistency. In the first step, it was ensured the data follows a normal distribution through a Shapiro-Wilk test. The results suggest that a normal distribution may be assumed. Visual inspection of the Q-Q plots corroborated this finding. Next, a check for outliers was performed, which did not indicate the presence of problematic cases. Levene’s tests exhibit variance homogeneity (baseline PSS-10: p = 0.077, post-intervention PSS-10: p = 0.166).

**Fig. 2:**
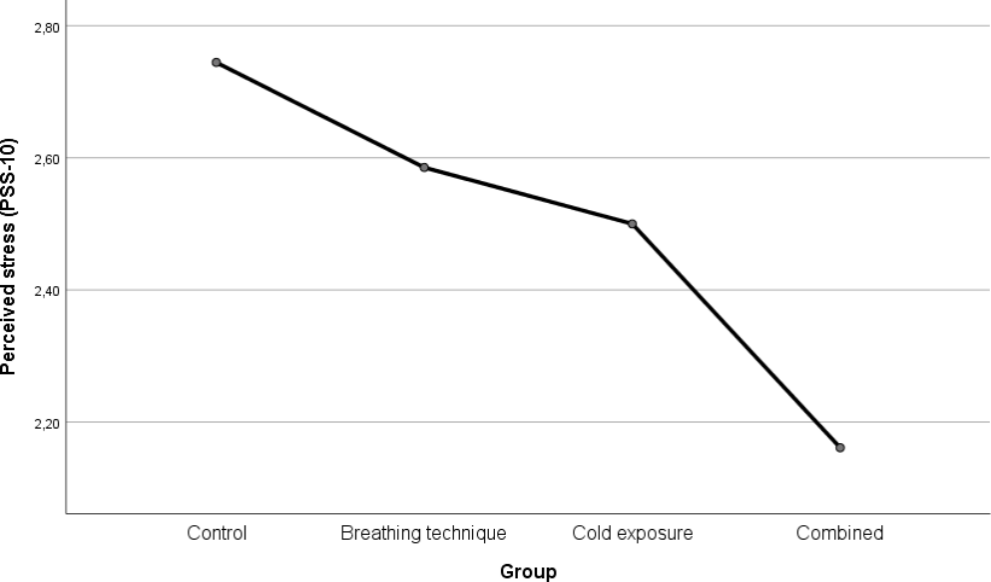
Results for PSS-10

Comparing post-intervention PSS-10, ANOVA results suggest an effect of the treatment: perceived stress was reduced, with the highest decrease found for the combined group (M = 2.16, SD = 0.45), followed by cold exposure (M = 2.50, SD = 0.77), and the breathing technique (M = 2.58, SD = 0.55). The control group reported the highest level of perceived stress (M = 2.74, SD = 0.59). The degree of perceived stress yielded statistically significant differences between the groups: F(3, 82) = 3.345, p = 0.023. The overall effect size was calculated using ω² and η², yielding 0.075 and 0.109, respectively. This may be considered a medium effect. Tukey’s post hoc analysis further revealed a significant difference between the combined group and the control group (0.583, p = 0.013, 95%-CI [0.09, 1.07]). Figure 2 presents the results for the PSS-10. Figure 2 presents the results for the PSS-10.

Groupwise analysis shows that perceived stress did not change over time for the participants in the control group (p = 0.332, partial η² = 0.038). However, significant effects were found for the breathing technique (p = 0.006, partial η² = 0.331), cold exposure (p = 0.008, partial η² = 0.291), and the combined application (p = 0.001, partial η² = 0.479), the latter of which yielded the largest effect.

The second analysis run evaluated the PSQ. Cronbach’s Alpha for the scale was 0.927, indicating a high degree of internal consistency. PSQ scores showed a substantial positive correlation with PSS-10 scores (r = 0.842, p < 0.001). Again, a Shapiro-Wilk test indicated normally distributed data, and a check for outliers did not detect problematic cases. Levene’s tests support the assumption of variance heterogeneity (baseline PSQ: p = 0.066, post-intervention PSQ: p = 0.134).

**Fig. 3:**
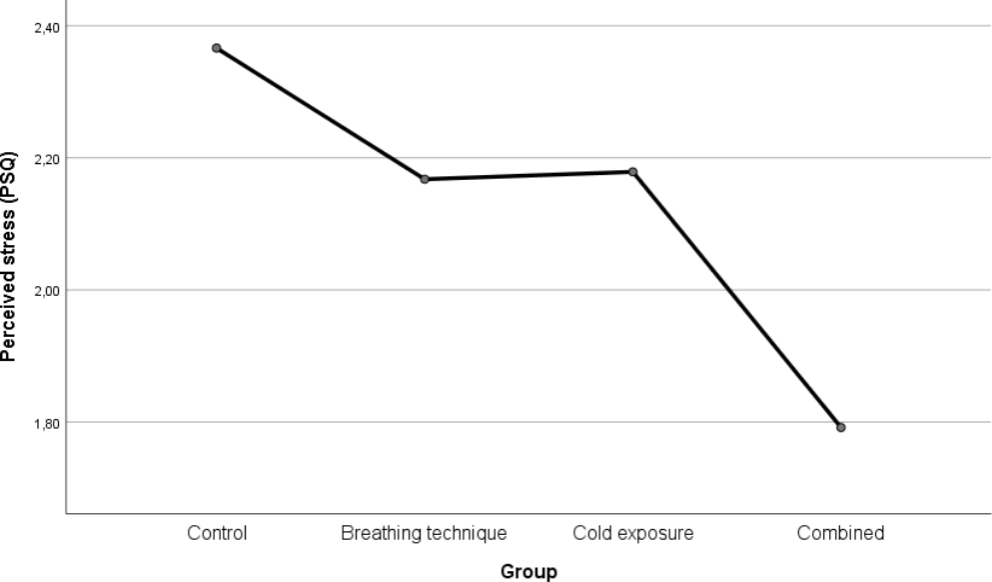
Results for PSQ

Again, the findings suggest an effect of the treatment: perceived stress was reduced, with the highest decrease found for the combined group (M = 1.79, SD = 0.33), followed by the breathing technique (M = 2.17, SD = 0.58), and cold exposure (M = 2.18, SD = 0.60). Consistent with the PSS-10 results, the control group reported the highest level of perceived stress (M = 2.37, SD = 0.50). Levene’s test suggests that homogeneity of variances may be assumed (p = 0.147). The degree of perceived stress yielded statistically significant differences between the groups: F(3, 82) = 4.321, p = 0.007. Effect sizes ω² and η² are calculated as 0.104 and 0.136, respectively, which may be considered reasonably large effects. Post hoc analysis (Tukey) again reveals a significant difference between the combined group and the control group (0.566, p = 0.004, 95%-CI [0.154, 0.995]). Figure 3 presents the results for the PSQ.

Investigating the change in perceived stress groupwise, the control group did not experience a statistically significant influence of time (p = 0.831, partial η² = 0.002), in contrast to the breathing technique group (p = 0.005, partial η² = 0.352), cold exposure (p = 0.012, partial η² = 0.263), and the combined group (p = 0.003, partial η² = 0.408). Hence, the effect was the most substantial for the integrated application of breathing and cold exposure techniques.

Both the PSS-10 and the PSQ agree in their assessment. Thus, H1 and H2 could be supported. The combined group integration both methods, yielded a significant reduction of perceived stress, corroborating H3.

## Discussion

Findings suggest that an application of the WHM is associated with a statistically significant decrease in perceived stress. While all exercise groups experienced this effect over time, the combined practice of a dedicated breathing technique and cold exposure yielded the highest benefit. Thus, the combination of both appears to have an additional, positive effect. This finding is consistent with the recommendations of the WHM, which proclaims the entanglement of their elements (Innerfire, 2021). Although post-intervention comparisons did not show significant differences for all groups in their post-hoc stress values but for the combined group and control group, this finding demonstrates the WHM’s potential for increasing individual mental well-being. Perceived stress values for the combined group decreased by 0.58 points for both the PSS-10 and the PSQ, which is a reduction by 21.17% (PSS-10) and 24.47% (PSQ), respectively, using the control groups’ values as a reference. A decline in perceived stress by one-fifth up to a quarter through two weeks of consecutive, non-pharmacological intervention may be considered a clinically relevant result.

Our study thus proposes to follow the combined approach, which is in line with physiological results reported in the literature: while breathing exercises and cold exposure yield benefits on their own, the implementation of a cold exposure practices was found to elevate the impact of breathing, and stand-alone cold exposure best alleviated symptoms in an endotoxemia trial (Zwaag et al., 2022). Hence, it appears that both elements complement each other. Previous findings suggest that this effect stems from a stress-induced analgesic, i.e., pain relieving, response (Brazaitis et al., 2014).

Regarding the measures for perceived stress – drawing on Figs. 2 and 3 – slight differences between PSS-10 and PSQ assessment may be observed. Previous research has found a strong but imperfect correlation between the two instruments (r = 0.73 for the initial 30-item version of the PSQ) (Levenstein et al., 1993). Thus, this discrepancy is viewed not to be an issue: in the context of our analysis, both instruments showed an even more substantial correlation of 0.842. Nevertheless, it is possible that the PSS-10 and the PSQ differ in the facets they capture considering individuals’ perceived stress, which might explain subtle deviations. For example, the factor structure of the PSS-10 has been a long-standing debate among scholars (see, e.g., Liu et al., 2020; Ng, 2013; Roberti et al., 2006). This difference may also explain the comparison between the breathing technique and the cold exposure groups in Figs. 2 and 3: while in Fig. 2, reporting results based on the PSS-10, participants practicing cold exposure report slightly lower values of perceived stress than those performing the breathing exercise, this small deviation is reversed for the PSQ, as shown in Fig. 3.

The combined approach, naturally, takes more time than the isolated exercises. Future research may thus examine whether longer training periods or intensified exercises (e.g., lower temperatures or longer duration regarding the cold exposure, increased numbers of inhalation cycles, more rounds and longer retention periods for the breathing technique) can lead to an equivalent outcome. Similarly, it would be interesting to reduce the extent of the combined practice to save time and make the WHM more accessible.

As the literature review shows, the WHM bears similarities to yoga-specific techniques such as pranayama. Future research may compare the effects of different types of exercises to gain insights into the most effective combination. Besides, further investigation may conduct the WHM for a longer period to examine potential effects of the breathing technique and the cold exposure, respectively.

## Conclusion

The WHM may be employed to reduce individuals’ perceived stress. Combined breathing techniques and cold exposure were found to yield the largest positive impact. As the WHM draws on techniques known from other contexts such as yogic pranayama breathing or cold-water exposure in hydrotherapy, our findings may also serve as additional evidence for their fruitfulness. Individuals seeking to reduce their perceived stress may seek to apply exercises that consider both forms of hormetic stress, i.e., breathing techniques and cold exposure in combination. In conclusion, our investigation shows that exercises constituting the WHM (and that are also being used in a similar fashion in traditional schools of mindfulness) are simple yet effective means to improve mental well-being that are applicable for a wide audience.

## Limitations

As for all scientific studies, limitations need to be addressed. First, inclusion criteria were relaxed, as participants of legal age that do not meet exclusion criteria were eligible. Second, the experiment was conducted over two weeks. While extant research has employed similar schedules, future investigations may examine the long-term impact of the individual and combined techniques. Besides, perceived stress was selected as the dependent variable. This decision was made in line with the WHM’s goal to improve personal well-being. Further insights are needed to link these results to objective criteria such as stress-induced symptoms and disease to illuminate the method’s potential influence on public health.

In the study at hand, all participants were asked to follow the same moderate breathing pattern. Changes in pace may alter the outcome: while slow breathing may induce vagus nerve stimulation (Brown & Gerbarg, 2005), a faster pace is assumed to “provide mild sympathetic stimulation” (Jerath et al., 2015). There is evidence that slow breathing yields more substantial benefits (Mourya et al., 2009). Some practices also combine both types (Zope & Zope, 2013), which might be a starting point for further investigation of the WHM and potential modulations. Ultimately, associations identified using statistical analysis are correlations. While related work renders the assumption of a causal relationship between the WHM and reduced perceived stress plausible, this interpretation needs to be treated cautiously.

## Data Availability

The datasets generated during and/or analysed during the current study are available from the corresponding author on reasonable request.

## Appendix A: PSS-10 items (translated from German)

**Table:**

PSS-10-1	In the last two weeks, how often have you been upset because of something that happened unexpectedly?
PSS-10-2	In the last two weeks, how often have you felt that you were unable to control the important things in your life?
PSS-10-3	In the last two weeks, how often have you felt nervous and “stressed”?
PSS-10-4	In the last two weeks, how often have you felt confident about your ability to handle your personal problems?
PSS-10-5	In the last two weeks, how often have you felt that things were going your way?
PSS-10-6	In the last two weeks, how often have you found that you could not cope with all the things that you had to do?
PSS-10-7	In the last two weeks, how often have you been able to control irritations in your life?
PSS-10-8	In the last two weeks, how often have you felt that you were on top of things?
PSS-10-9	In the last two weeks, how often have you been angered because of things that were outside your control?
PSS-10-10	In the last two weeks, how often have you felt difficulties were piling up so high that you could not overcome them?

## Appendix B: PSQ items (translated from German)

**Table:**

PSQ-1	You feel rested
PSQ-2	You feel that too many demands are being made on you
PSQ-3	You have too many things to do
PSQ-4	You feel you’re doing things you really like
PSQ-5	You fear you may not manage to attain your goals
PSQ-6	You feel calm
PSQ-7	You feel frustrated
PSQ-8	You are full of energy
PSQ-9	You feel tense
PSQ-10	Your problems seem to be piling up
PSQ-11	You feel you’re in a hurry
PSQ-12	You feel safe and protected
PSQ-13	You have many worries
PSQ-14	You enjoy yourself
PSQ-15	You are afraid of the future
PSQ-16	You are lighthearted
PSQ-17	You feel mentally exhausted
PSQ-18	You have trouble relaxing
PSQ-19	You have enough time for yourself
PSQ-20	You feel under pressure from deadlines
